# From Micro to Macro: The Combination of Consciousness

**DOI:** 10.3389/fpsyg.2022.755465

**Published:** 2022-03-31

**Authors:** Asa Young, Isabella Robbins, Shivang Shelat

**Affiliations:** Department of Psychological and Brain Sciences, University of California, Santa Barbara, Santa Barbara, CA, United States

**Keywords:** resonance, altered states of consciousness, interpersonal synchrony, hyperscanning, human mind, social interaction, neural synchronization

## Abstract

Crick and Koch’s 1990 “neurobiological theory of consciousness” sparked the race for the physical correlates of subjective experience. 30 years later, cognitive sciences trend toward consideration of the brain’s electromagnetic field as the primary seat of consciousness, the “to be” of the individual. Recent advancements in laboratory tools have preceded an influx of studies reporting a synchronization between the neuronally generated EM fields of interacting individuals. An embodied and enactive neuroscientific approach has gained traction in the wake of these findings wherein consciousness and cognition are theorized to be regulated and distributed beyond the individual. We approach this frontier to extend the implications of person-to-person synchrony to propose a process of combination whereby coupled individual agents merge into a hierarchical cognitive system to which they are subsidiary. Such is to say, the complex mammalian consciousness humans possess may not be the tip of the iceberg, but another step in a succeeding staircase. To this end, the axioms and conjectures of General Resonance Theory are utilized to describe this phenomenon of interpersonal resonant combination. Our proposal describes a coupled system of spatially distributed EM fields that are synchronized through recurrent, entraining behavioral interactions. The system, having achieved sufficient synchronization, enjoys an optimization of information flow that alters the conscious states of its merging agents and enhances group performance capabilities. In the race for the neurobiological correlates of subjective experience, we attempt the first steps in the journey toward defining the physical basis of “group consciousness.” The establishment of a concrete account of the combination of consciousness at a scale superseding individual human consciousness remains speculation, but our suggested approach provides a framework for empirical testing of these possibilities.

## Introduction

The Borg of *Star Trek*, Xenomorphs of *Alien*, and Wights of *Game of Thrones* (*A Song of Ice and Fire*) are single conscious entities united across multiple individual bodies often used as a foil to the protagonists’ flamboyant individualism. These science fiction terrors constitute a group in which constituent members engage in a combined, supervening consciousness. The horror of the “hive mind” stems from the undying loyalty, lockstep initiative, and the abolition of self that accompanies being one with the collective. A permeated motif of cultural media (O’Sullivan, 2010), hive minds, or extended consciousness is a topic frequently engaged yet remains shrouded in popular mysticism.

One such example of engagement with mystical undertones is the Global Consciousness Project’s longstanding study of global consciousness and random number generation. Their report of the events of September 11th, 2001 gained notoriety when it described attenuation of random number variation as an effect of unified, global consciousness ignited by shared global attention on the terrorist attacks. Another example may be found in a question commonly posed to cognitive scientists: “Does the Internet, currently or in the near future, possess the capacity to constitute a conscious entity?” (O’Gieblyn, 2020; see also Hunt, 2014). The article concludes “Perhaps,” allowing the reader’s mind to linger on the possibility of a conscious brain to which they are akin to a single neuron. We appear to possess a popular, natural notion of combined consciousness or conscious entities at scales superseding our own.

The natural presupposition of combined consciousness is accompanied by a parallel notion that groups of discrete individuals constitute Gestalts distinct from the sum of its parts. This parallel notion has become operationalized in experimental psychology through such concepts as intergroup emotion theory (Smith et al., 2007; Mackie et al., 2008) and collective intelligence factor (Woolley et al., 2010). Equipped with these recurring themes, we will explore combined consciousness primarily through the lens of cognitive neuroscience. Normally regarded as a fringe topic, we will attempt to explore the possibility of its occurrence while remaining within the confines of accepted scientific method. We ask only that the reader entertains (without endorsing) the possibility of consciousness at scales beyond the individual human level, as we sketch an outline of how this may physically occur, how it may be measured, and what it implies for our understanding of conscious, cognitive systems (Schooler et al., 2018).

There has been a growing trend in the cognitive sciences to look to rhythmic neural oscillations of neuronally generated electromagnetic fields (Anastassiou et al., 2011; Buzsáki et al., 2012; Hales, 2017; Chiang et al., 2019) as the primary seat of consciousness (McFadden, 2013; Jones, 2016; Hunt and Schooler, 2019; Hunt, 2020). In this approach, scientists look to the “oscillatory correlates of consciousness” as the primary physical dynamic for tracking the presence and complexity of consciousness. It is suggested that the dynamics of consciousness may be identical to the dynamics of the various EEG frequency bands from slow oscillations (below 0.2 Hz), to delta (0.2–3 Hz), theta (3–8 Hz), alpha (8–12 Hz), beta (12–27 Hz), through gamma (27–100 Hz), and higher (Fields, 2020).

Not only are these rhythms known to be important dynamics of individual consciousness; over the last two decades, research has revealed that the oscillatory correlates of distinct individuals will often synchronize with the neural oscillations of other individuals of the same species. This phenomenon, coined inter-individual neural synchrony (also referred to as inter-brain coupling, inter-brain synchrony, interpersonal brain coupling, etc., abbreviated here to INS), is a product of shared, recurrent stimulus-to-brain coupling, amidst joint action behavior, whereby the stimulus is produced by the brain of one individual, conveyed by the motor system, and received by the other’s sensory system (Hasson et al., 2012). The correlates of joint action are observed at a young age, facilitating social development in children. Infants and their parents/caregivers engage in dyadic activity by utilizing a common visual focus to express shared intentionality (Mundy and Newell, 2007). Both infant and caregiver use this common visual focus to establish a point of reference, aligning their mutual attention to the stimulus (Lachat and George, 2012). By proxy of this shared entrainment, infant and caregiver synchronize their neural activity (Leong et al., 2017).

Inspired by the General Resonance Theory of consciousness (Hunt and Schooler, 2019; Hunt, 2020), we will develop a model of interpersonal resonant combination (a broader framework for INS) that builds upon and extends the implications of joint action findings. This paper attempts to integrate empirical findings of synchronization in the oscillatory correlates of consciousness with GRT’s generalized resonance principles of micro-to-macro combination. It is our hope that this framework can extend what is currently only speculation about higher scales of consciousness than the individual scale into a testable framework. It will be stressed here that this is an application solely of General Resonance Theory’s framework as there are dissenting opinions, as will be reviewed below, regarding the parameters of extended or combined consciousness.

## Intra-Body Resonance to Inter-Individual Synchrony

GRT postulates that all matter resonates (the resonance axiom) and that all matter possess, at the very least, a rudimentary capacity for consciousness (the panpsychism axiom). Resonating structures will, when in sufficient proximity to influence one another (Hunt, 2020), resonate at the same frequency with other proximal matter and establish a shared resonance frequency (the coupling axiom). The shared resonance achieves a distinctive phase transition in the speed and/or bandwidth of information processing between the resonating constituents, thereby generating a larger, more complex physical system that has an increased capacity for conscious experience (the shared resonance conjecture). Shared resonance between coupled oscillators, specifically between neuronally generated EM fields (and the information processing made possible with EM fields), is, GRT suggests, necessary and sufficient for mammalian consciousness.

The Huygens clock phenomenon provides an effective illustration of the ontological foundations upon which the remainder of this article will build from. Christiaan Huygens, inventor of the pendulum clock, serendipitously observed that his clocks will, when sharing a medium (physical connection *via* floor or wall), synchronize pendulum swings regardless of their starting position. The one-second pulses of the clock’s internal timekeeping mechanisms reverberate through the shared medium to influence the partner clock and, within a sufficient period of time, this bi-directional flow of one-second pulses will couple the two together and synchronize swing cycles (Oliveira and Melo, 2015). This shared resonance between oscillators or systems of oscillators is the mechanism by which micro-conscious entities combine into a new macro-conscious entity. Our micro and macro designations, that will appear often throughout this article, are relative terms used to designate pre- and post-combination units. The two clocks in the above example, in their incorporation into the coupled oscillator system, are micro-systems (clock) that are nested in the supervening macro-system (clock-clock). As we take another step up the staircase of combination, the micro prefix will designate the clock-clock system in their coupling to a partner clock-clock system to form a new macro: group-group system and so on. With each step, though, the system transitions through spatiotemporal scales. We will return to this point following the summary of the brain–body spatiotemporal hierarchy, upon which this current description of interpersonal resonant combination is derived.

As matter evolves into more complex forms so does its capacity for consciousness. The mammalian body displays a number of these shared resonance interactions between the central brain and the organs of the periphery (Young et al., 2022). The oscillatory links merge the distant neural clusters into a unified whole, thereby producing the complex mammalian consciousness that we now enjoy, as a product not only of intra-cerebral shared resonance but also whole-body shared resonance of different types. The three extra-cerebral shared resonances that will briefly be reviewed seem to play functional roles in regulating and distributing cognitive activity throughout the Gestalt organism.

The gastric basal rhythm, a 0.05 Hz frequency emitted by the peristaltic organs, couples to the brain’s alpha frequency forming a gastric-brain shared resonance interaction, as measured by a combination of EEG (electroencephalogram) and EGG (electrogastrogram; Richter et al., 2017; Rebollo et al., 2018). This comprises one link in the oscillatory arm of the gut-brain-axis that resonates with the brain to maintain homeostasis and interoceptive sense (Huizinga, 2017; Jena et al., 2020). Within the brain, the BOLD (blood oxygen level-dependent) signal also shares this extra-cerebral resonance, maintaining a positive relationship with the gastric basal rhythm (Rebollo et al., 2018).

Cardiac-brain shared resonance is established through the coupling of the 0.1 Hz heart resonant frequency, a distinctive high amplitude peak in the HRV power spectrum, to the brain’s alpha and beta frequencies (McCraty et al., 2009). The resonance relationship functions as a regulator of emotional experience. Coupling is established in post-traumatic stress disorder (PTSD) patients undergoing Somatic Experiencing trauma therapy when a threat response is successfully re-negotiated (Whitehouse and Heller, 2008).

The saccadic rhythm, generated at 3–4 Hz, underlies the retinal-brain shared resonance relationship with cerebral theta and alpha waves (Leszczynski and Schroeder, 2019; Leszczynski et al., 2021). The endogenous rhythm aligns the neural oscillations to the flow of incoming visual information. Retinal-brain shared resonance facilitates our active visual sensing of the external environment.

These shared resonance interactions comprise distinct neural clusters synchronizing and contributing to the larger merged system: the organism. Through a GRT lens, the observer can denote the neat nesting of many micro-conscious combinations that generate the complex, unified human or animal thereof (Young et al., 2022). Each of these merged systems can be defined by the slowest shared resonance (SSR), the GRT principle postulating that the boundary of a conscious system is defined by the slowest common denominator frequency by which shared resonance occurs (the boundary conjecture). The 0.05 Hz gastric basal rhythm defines the gastric-brain system, the 0.1 Hz heart resonance frequency defines the cardiac-brain system, and the 3–4 Hz saccadic rhythm defines the retinal-brain system. The spatialization of consciousness across the distributed nervous system implies a mechanism of information integration across spatiotemporal scales. Cross-frequency coupling (CFC), the modulation of a higher frequency by one that is lower (Canolty and Knight, 2010), represents the synchrony architecture through which the sub-hertz rhythms of the body’s organs may influence the higher frequency bands of the EEG. As the distribution of the coupled system increases, the unifying consciousness that encompasses it is constrained by the increasingly slower frequencies that underlie it.

This model can be rearticulated to fit our needs in exploring INS combination. Hyperscanning EEG (Liu et al., 2018; Czeszumski et al., 2020), fNIRI (Scholkmann et al., 2013), and fMRI (Montague et al., 2002) are recent laboratory tools that have made possible the recording of phase synchronization of neural rhythms across groups of individuals. Briefly, the hyperscanning technique simultaneously measures activity between two or more brains for identifying commonalities (Montague et al., 2002). The establishment of such synchrony relies on two factors: mutual attention (Hasson et al., 2012; Koban et al., 2019; Nguyen et al., 2020; Djalovski et al., 2021) and the presence of prior empathetic relationships (close friends, true couple, etc.) within the participant pool (Kinreich et al., 2017; Djalovski et al., 2021). We propose, in line with Hasson et al. (2012), that INS is defined by a shared entraining stimulus. Entrainment, the (mostly) unidirectional coupling of one oscillator to another (Lakatos et al., 2019), to a shared behavioral rhythm facilitates the alignment of neural activity within the entrained group.

The coupled system is defined and limited by the slowest shared resonance to which all group members are entrained. Slow frequencies traverse spatial distances at greater speeds, allowing information to arrive and be incorporated into the oscillatory cycles of constituent group members (Dehaene, 2014). The shared entraining signal, within our model, serves as the SSR of the interpersonal cognitive system. The INS-SSRs we have identified are of ultraslow variety, with the exception of speech, and thus possess the capacity to traverse the spatial distance between group members for their entrainment.

The synchronization between neural clusters of distinct brains can be accomplished via a substitution of direct axonal connections with shared exogenous entrainment of neural rhythms to identical effect. The ultraslow entraining oscillations that inaugurate INS then define the coupled group. Our compiled list of INS-SSRs will be discussed in the following section.

The experience, objective (i.e., task performance), and subjective (self-other merging, feelings of affiliation, etc.) of individuals as they synchronize and become nested in the larger group cognitive system is very much altered from their regular waking consciousness. Some authors have called this experience “group flow” (Kotler and Wheal, 2018). GRT’s framework assumes continuation (no extinction) of micro-conscious entities as they merge into a macro-system (the nested consciousness conjecture). Although it is far too early and there is not sufficient evidence to definitively claim the presence of a macro-conscious system, a “group consciousness,” within the entrained, synchronized group, we will put forth the proposition that the inter-individual resonance in the oscillatory correlates produces the possibility of such a claim. If it is to be measured, it will likely be through the mapping of the altered states of consciousness in the unextinguished micro-conscious individuals pre-, peri-, and post-combination. The penultimate section will review several feasible experimental paradigms for testing our proposition. This approach must suffice until a proper “psychometer” (Hunt et al., 2021) may be developed for measuring a phenomenal consciousness more complex than our own.

## Inter-Individual SSRs

We have compiled a list of INS-SSRs that possess the entraining capacity to serve as the coupling signal underlying synchronized groups. All of these are of social variety in that they may originate from constituent group members to which sender and receiver/s may share in coupling. The coupling signal is dependent on the group’s focus of attention and can be transferable in this respect. As aggregate attention on one shared rhythm wanes and is reignited upon another oscillator, the SSR demarcation is thus transferred. Our list of INS-SSRs includes syllabic rate of speech, respiratory cycles, behavioral cue exchange, and interpersonal sexual activity (shared tactile stimulation). These entraining modalities are conveyed at ultraslow frequencies, with exception to speech which is conveyed at slow frequencies. There is some discussion regarding “true” versus “false” interpersonal synchrony (Valencia and Froese, 2020). Reciprocal adjustment of ongoing rhythms is regarded as necessary for true synchrony as opposed to the driving of rhythms by an external source. The behavioral nature of the entraining signals listed below, although external drivers, exhibits a reciprocal adjustment between attending group members (Dumas et al., 2010; Woolley et al., 2010; Nguyen et al., 2020; Loh and Froese, 2021) and thus, in our view, constitute a true synchronization.

Human speech is expressed at the syllabic rate of 3–8 Hz (Fujii and Wan, 2014). In attending speech, the listener is entrained in delta, theta, and gamma waves (Giraud and Poeppel, 2012; Gross et al., 2013). The speaker is entrained in delta, theta, and alpha waves (Pérez et al., 2017). INS is observed between interlocutors contingent upon the listener’s possession of the speaker’s language (Spiegelhalder et al., 2014). Speech as an SSR appears as the most intuitive of our formative list. The group, as it attends a speaker, is entrained and synchronized to the speaker and, by proxy of the shared entrainment, to the rest of the aggregate. With communicative turn taking, a staple element of interpersonal communication (Woolley et al., 2010; Nguyen et al., 2020), the SSR demarcation will remain transferable between speakers.

In a singular-brain frame of reference, nasal respiration, occurring at 0.16–0.33 Hz, entrains delta, theta, and beta EEG bands (Zelano et al., 2016). Respiratory cycles are theorized to constitute a global coupling rhythm by which complex neural activity in the brain is organized (Başar, 2008; Corcoran et al., 2018; Klimesch, 2018). On a multi-brain scale, group entrainment can occur to the breathing frequency of an attended individual (Bachrach et al., 2015). In the cited example, a dancer, the subject of group attention, is performing a routine characterized by incredibly slow movement and centered around the dancer’s respiratory cycles. A positive correlation was observed between audience attention and interpersonal respiratory synchrony. Coupled respiration may serve as a group entraining rhythm and represents the most feasible option for empirical testing. Coupling participants *via* respiration is unobtrusive and allows experimenters to incorporate a task amidst coupling, opening the analysis to include synchronization as a factor as opposed to an effect.

Behavioral cue exchange is represented by the sender’s ostensible cues that indicate communicative intent and the receiver’s contingent responsiveness that implies communicative sensitivity (Wass et al., 2020). As group attention shifts, the roles of imitator (receiver) and sender are regularly exchanged, and mutual behavioral negotiation engaged (Dumas et al., 2010). This behavioral dialog generates a state of behavioral synchrony within communicating groups. Ostensive cues in particular increase the behavioral entrainment of the receiver to the sender (Feldman, 2007; Murray et al., 2016; Wass et al., 2020). Participants entrained in behavior cues exhibit inter-brain synchronization in alpha-mu, beta, and gamma bands (Dumas et al., 2010).

Sexual activity, interpersonal, or solitary, occurring at a semi-stable rhythm, offers an avenue for entrainment in producing orgasm, hypothesized to be a trance state enabled by the entrainment (Safron, 2016). Interpersonal sexual activity should predictably exhibit INS during a portion in which both (or more) partners are simultaneously being stimulated by a shared rhythm (i.e., penetration or other kinds of stimulation). Surprisingly, no study has looked to INS between active sexual partners during performance (to our knowledge at the time of this writing). Although this modality is empirically less dense than our other entraining avenues, it represents a promising line for future research.

Exogenous entraining stimuli of a non-social variety present a point of contention with regard to true versus false synchronization. The brain is liable to be entrained *via* photic (Adrian and Matthews, 1934) and auditory (Chatrian et al., 1960) means, collectively termed audiovisual entrainment (AVE). Music, present in nearly half (44%) of experience-sampled events (Sloboda and O’neill, 2001), is a reliable entraining stimulus of cortical oscillations (Doelling and Poeppel, 2015). On an interpersonal scale, music comprises a coupling signal that is a fundamental component of social coordination and cultural practices (Clayton et al., 2020). INS within synchronized groups conducting coordinated actions is strengthened by the administration of steady exogenous auditory rhythms (Ikeda et al., 2017). The Clayton piece would argue that music, an external driver, is a viable coupling signal for group practices and would reflect a true interpersonal synchronization. It may be argued that the necessary mutual adjustment is present between musicians in producing the stimuli as well as between musicians and audience in situations wherein the audience is considered an active participant in the performance. The Ikeda piece takes a lighter stance on their findings in that the exogenous stimuli during group walking (activity that exhibits INS) was an adjunct that strengthened prior synchronization. One method of artificial synchronization, shared transcranial brain stimulation (TBS), bypasses the sensory pathways entirely to directly entrain neural oscillations. Mice, prepared with optogenetics, displayed greater affinity when TBS signals were synchronized than when each was stimulated at different frequencies (Yang et al., 2021). This novel method of neural coupling muddies the distinction between true and false synchronization, and any attempt here to define it would rely purely on intuition. Artificial oscillators possess the potential to constitute a group coupling signal or, at the very least, a facilitator of synchronization by social means.

This list is by no means exhaustive. There exists a large conceptual space for the addition of countless other group entraining stimuli, as they are discovered and elucidated. Speech, respiration, behavioral cues, and sexual stimulation are the most readily available modalities of social origin at the time of our writing. This line of research is one deserving of additional attention and development. The following section will review qualitative and quantitative markers that distinguish a truly synchronized, merged group apart from one exhibiting spurious synchrony.

## Phase Transition

GRT micro-to-macro combination is marked by a phase transition in the informational speeds between micro-conscious entities as a result of shared resonance (Hunt and Schooler, 2019). This is a term borrowed from physics referring to a process in which a critical threshold is exceeded, and the state of information flow is modified. The phase transition that occurs within the conscious mammalian brain, according to GRT, is the shift from electrochemical information (synaptic) to electromagnetic field exchanges (ephaptic), the latter of which is significantly faster. This point of criticality supports the vast flow of information during conscious, as opposed to unconscious, states (Toker et al., 2022). As informational speed and bandwidth increase, the depth and scope of the phenomenal consciousness increase. Within this section, “phase transition” will revert to an analog representation of its meaning to refer to a (as yet unknown) critical point that, when exceeded, is then followed by a collection of cognitive, social, and behavioral changes in synchronized groups as a result of optimized information flow. Returning to the Huygens’ clock illustration, the transition from a desynchronized to a synchronized state, within which collective oscillations emerge, occurs when the system exceeds coupling strength K (Kuramoto, 1975).

Although the GRT framework suggests the presence of a larger macro-consciousness that encompasses a sufficiently synchronized group, this is a difficult position within the current state of cognitive neuroscience. A point of contention among field theorists includes whether decreasing field strength across space limits the ability of EM fields to unify consciousness (see Libet, 1994, Jones, 2013, and McFadden, 2013 for dissenting opinions). This section, however, will examine evidence that likely represents a phase transition in-group information processing and may indicate some kind of larger group consciousness, albeit fleeting. Our purview is purely in building a representative model of what micro-to-macro combination of superseding scale would look like according to GRT axioms and conjectures.

In synchronizing with local group members, the brain’s internal representations of the self and the other become blurred, optimizing predictive capabilities and efficiently increasing cooperative capacity (Koban et al., 2019). This is a result of temporally aligning the oscillatory windows of group members, a phenomenon akin to the saccadic entrainment of theta and alpha EEG bands during active sensing (Leszczynski and Schroeder, 2019; Leszczynski et al., 2021). The temporal alignment of oscillators generates a coupled system within which the rhythmic flow of visual information is received in-phase of visual cortical oscillations. Perceptual thresholds are lowered, and acute visual sense is facilitated. A similarly effective coupled system emerges within a synchronized group that exhibits an identical alignment of oscillatory windows.

We have compiled a list of cognitive effects that appear representative of the occurrence of phase transition across individuals. Group synchrony is, for example, associated with:

Increased performance in cooperative tasks (Szymanski et al., 2017; Nguyen et al., 2020). It is a robust finding that INS-established groups experience greater cooperative task performance as compared to non-synchronized groups. The cited Szymanski study compared individual performance against non-synchronized groups against synchronized groups on identical tasks. Synchronized groups exhibited significant increases in task capability.
Self-other merging (Valencia and Froese, 2020). This is one of several signs that there is some manner of altered state of consciousness induced during group synchronization (perhaps a bit on the nose). It falls within Ludwig’s ASC framework under characteristic E: “body image change” (Ludwig, 1966). Hyperscanning fMRI studies indicate a shared synchronization in the right anterior insula, a structure responsible for interoception (Koike et al., 2019; Yoshioka et al., 2021). Tied to this is the hypothesized increased capacity for empathy regarding group members (Hove, 2008), an intuitive result of experienced self-other merging.
Increased feelings of group affiliation (Hove and Risen, 2009; Hoehl et al., 2021). There is a positive relationship between group affiliation and INS. Group affiliation increases the likelihood of INS establishment. Inversely, INS generates stronger feelings of inclusion in the group. Synchronous groups, experiencing cues to their affiliation, exhibit behavior that is measurably more prosocial (Hu et al., 2017; Cirelli, 2018), an effect convergent of both factors. Additionally, INS may influence intergroup relations: individuals are more strongly synchronized to politically like-minded peers during polarizing political debate than to political opponents. The strength of synchrony between committed partisans is associated with producing similar polarized attitudes regarding the debate (van Baar et al., 2021).
Increased capacity for interpersonal communication (Miles et al., 2009; Hoehl et al., 2021). The coupling of neural activity between communicators aligns predictions with outputs. Wilson suggests that coupled groups in coordination experience an ease of information flow stemming from their synchronization that saves computational power (Wilson, 2001). Friston and Frith (2015), in tandem with Wilson, propose that, to avoid an infinite regression of modeling your partner that is modeling you, an n + 1 “shared model/prediction” emerges to unify the active inference mechanisms of interacting agents.

The coupling of neural activity across a group enables an optimized synchronization of cognitive-behavioral inputs and outputs across the aggregate (Koban et al., 2019; Valencia and Froese, 2020). Just as theta and alpha entrainment by saccadic rhythms decreases perceptual thresholds for acute visual sense, INS increases sensitivity to the conspecific nodes of the emerging group brain. The improvement of cooperative ability and altered experience of the individual demonstrates a phenomenon of integration.

In our discussion of resonance at the inter-individual scale, effects such as those listed are hypothesized to signal a critical point by which the collection of individuals becomes an integrated, functional unit. The exact moment the critical point is traversed is yet unknown. The INS effects only suggest the line was likely crossed at some moment preceding its social and cognitive influences. Thus, INS separates a synchronized aggregate from a mere aggregation of individuals.

## Interpersonal Resonant Combination

Twenty years of brain-to-brain synchrony literature has informed us of the peculiarities that emerge amidst intensive social interaction. The collection of effects is accompanied by the growing notion that it is not an epiphenomenal occurrence. Arising in early developmental stages, it facilitates social learning between child and caregiver. The phenomenon is rearticulated to other social interactions to function as a neural bind between communicators, cooperators, and companions. As we approach this advancing frontier, it is time we begin considering the implications of brain-to-brain synchronization.

The mechanism of combination that unifies the central and peripheral nervous systems is reflected in the similar resonance that occurs between distinct brains of cooperative group members. It is a recent hypothesis that INS may be the means by which micro-to-macro combination of interpersonal scale occurs (Valencia and Froese, 2020). Valencia and Froese’s recent review of similar interpersonal EEG synchrony, in light of Clark’s “extended mind framework” (Clark and Chalmers, 1998), hypothesized the origins of an extended consciousness rooted in the interpersonal synchronization of the fastest EEG frequency bands. Aligning the quantitative neural oscillations associated with qualitative experiences, when paired with various effects of social closeness and cooperation, makes such conclusions of possible combination or consciousness extension relevant.

We echo this hypothesis under the auspices of GRT’s axioms and conjectures. In line with previous research, we support the notion that INS is established *via* shared neural oscillations, entrained through behavioral rhythms that arise during social interaction and selected for by group attention. Utilizing GRT’s SSR conjecture, we suggest that the slowest shared entraining signal be designated as the boundary of the macro-cognitive system that encompasses the synchronized group.

At the extreme end of our speculation, we hypothesize the macro-cognitive system that has succeeded in a phase transition in group information exchange constitutes a macro-conscious entity to which constituent group members are part and parcel. Within the GRT framework, merged group members are not extinguished as merged micro-conscious entities but continue to persevere as individual and contributing agents. This, however, does not imply mutual horizontal access to the phenomenal contents of synchronized group members (i.e., shared consciousness). Instead, our description is that of a combined consciousness in which interpersonal synchronization of the individual oscillatory correlates of consciousness may link, horizontally, conspecific nodes that combine, vertically, into a supervening n + 1 system. The supervening system exhibits a greater (the contents of all merged agents) and deeper (increased horizontal communicative capacity) processing of information, the process and products of which are distinct from its merged agents. The combined system will span multiple spatiotemporal scales. Ultraslow behavioral rhythms entrain EEG bands and likewise affect other disparate neural rhythms that functionally interact with the entrained frequency. Akin to the brain–body spatiotemporal hierarchy referenced in section “Intra-Body Resonance to Inter-individual Synchrony”, the group consciousness is constrained by the phase state of the recurrent interactions that uphold it (Loh and Froese, 2021).

The notion that groups are entities distinct from the constituent individuals was referenced in the introduction as a common motif in popular knowledge and an increasingly operationalized factor in recent psychological studies. This text details the neuroelectrical backbone upon which such a notion may exist. The following will detail several empirical concepts we would speculate to be products of a supervening cognitive system. Intergroup Emotion Theory describes an emotional system originating within the social group that governs intergroup relations and perceptions (Mackie et al., 2008). In-group INS is associated with out-group hostility (Yang et al., 2020) and the strength of neural synchrony between like-minded partisans influences their perceptions of a prior conflict with political opponents (van Baar et al., 2021). Collective intelligence factor, a measure of group capability apart from individual performance, is correlated with social sensitivity and communicative turn taking (Woolley et al., 2010). These are components involved in the generation of INS: active, attending individuals and reciprocal adjustment of behavior. The previous section discusses improved group performance in cooperative tasks as a robust finding in hyperscanning studies.

In considering what constitutes the phenomenal experience of the macro-system, we may respond by regarding the influences discussed in the previous section as components of the group experience. As a nested yet unextinguished agent, the individual will experience an alteration in their state of consciousness: self-other merging and increased feelings of affiliation toward synchronized group members. The macro-system, possessing the contents of merged agents and optimized communication between its nested nodes, exhibits an information processing that increases performance capability and a cognitive influence in separating in-group (merged) and out-group (non-merged). To unlock the deeper contents, the “to be” so to speak, of merged/merging agents and the system they nest within, which we theorize is a conscious entity in and of itself, we suggest the mapping of altering states of consciousness as individuals combine through INS. Variation in the integrating brains and individual subjective experience will likely provide clues to the hypothesized macro-consciousness they are nested within, however temporary such nesting may be. The following section will propose three feasible experiments for such testing.

## ASC Mapping

We approach macro-consciousness through the contributing agents and aim to use data collected from the parts to make conclusions of the whole. As such, our proposed paradigms target shared experience and anomalous cognition associated with a supervening cognitive system. By sharing entrainment through oscillators discussed in section “Inter-individual SSRs”, INS will be imposed as a factor and ASC inducement, to varying degrees of severity, will be measured as the dependent variable. A mixed participant pool of stranger and true couple dyads is recommended in the following configurations for all three proposed studies: synched-stranger dyads, non-synched-stranger dyads, synched-empathetic dyads, and non-synched-empathetic dyads.

## Shared Experience of Time and Self-Other Merging

The first of our proposed experiments targets shared subjective experience of time and self-other merging. It is hypothesized that there will be a positive association between homogeneity in temporal experience and self-other merging with the INS factor. That is to say the strength of INS (closer to zero-lag synchrony) will correlate with increased homogeneity of temporal experience and increased perception of self-other merging. The self-other merging is an effect regularly associated with INS (Valencia and Froese, 2020). The qualitative experience of time is theorized to rely on the quantitative frequency of neural oscillations. This is supported by Samaha’s results suggesting the frequency of alpha oscillations underlie the temporal granularity of visual perception (Samaha and Postle, 2015).

Dyads will share entrainment *via* oscillators listed in section “Inter-individual SSRs”, although we suggest respiration coupling be implemented as the least demanding of the selection. As participants synchronize and are recorded through hyperscanning EEG, a self-other merging scale is administered intermittently, a method similar to mind wandering studies’ use of thought probes (Smallwood and Schooler, 2006). Following the synchronization task (coupled respiration), a scale measuring subjective experience of time is administered to which questions are answered relating to the time frame of coupling.

Possible measures are included here: the Inclusion of Other in the Self Scale (IOS) for self-other merging (Aron et al., 1992) and the Time Experience Scale (TES) for temporal experience (Sanders, 1986). The TES factor of interest is “Slow Tempo,” the subjective speed of time in a specific situation. If our hypotheses hold true, there will be a positive correlation between IOS results and INS, homogeneity of TES results and INS, and IOS results and homogeneity of TES results. Such results would demonstrate some manner of merged experience between synchronized group members.

## Hyperscanning Religious Rituals

INS is, in our suggested framework, contingent upon two factors: empathetic relationships and mutual attention. Religious group rituals performed by devout religious followers fulfill both requirements, but surprisingly no hyperscanning study has been published to fill this niche. The benefits of a hyperscanning study involving devout religious followers engaging in ritual are 2-fold: (1) this will be the first hyperscanning study in this domain and (2) a correlation may be established between the experience of God (or related deity) and strength of INS.

We hypothesize the presence of INS within such a group. Difficulty will arise in the equipment’s interference with the ritual. The headgear may be intrusive to our preferred natural setting. Portable EEG, used in Dikker’s classroom study (Dikker et al., 2017), may alleviate these issues (Debener et al., 2012).

Contingent on the first hypothesis’ successful results, we hypothesize the experience of God, equated to the religious experience itself (Alston, 2014), will be associated with INS establishment. Scales for God experiences are rare and often not validated. We suggest an inventory of select items from validated mystical experience scales such as Taves’ Inventory of Non-Ordinary Experiences (Taves et al., 2019). The selected inventory should contain items measuring emotional, sensory, and non-ordinary experiences intuitively and empirically associated with God. Empirically supported items include emotional measures for positive affect, peace, joy, and unconditional love (Beauregard and Paquette, 2008). Intuitive items include feelings of a non-ordinary presence, absorption, and connectedness to others.

The experience of God is selected as one such manifestation/interpretation of a group macro-conscious entity and a hypothetical ASC of combination. The implications of significant effects for the described study establish a preliminary link between INS and an experience of a supervening entity. The philosophical implications for such doctrines as Whitehead’s panentheism (Griffin, 2007, 2008) would require a separate article entirely.

## Interpersonal Synchrony in Physiological Rhythms

The shared resonance relationships between the peripheral organs and central nervous system covered in section “Intra-Body Resonance to Inter-individual Synchrony”, the predecessor model to interpersonal resonant combination, constitute the oscillatory links of an embodied mind (Young et al., 2022). The body constrains, distributes, and regulates cognitive processes (Foglia and Wilson, 2013) allowing for a theoretical extension of consciousness to an organism-wide phenomenon. The previous two experimental routes suggest various measurements of cerebral activity and their association with merging experience. Here, we recommend the third avenue through the hyperscan recording of physiological synchrony across empathetic dyads engaged in joint action.

Interpersonal synchrony research has taken a recent turn in this direction. Current work utilizes physiological variables in addition to behavioral and neural (Mayo and Gordon, 2020, see also Helm et al., 2018). Variables include, for example: heart rate, heart rate variability, respiratory sinus arrhythmia, cardiological impedance, body tremor, blood pressure, and electrodermal activity. The spontaneous synchronization of these endogenous rhythms, within an embodied framework, represents an extension to our INS model of resonant combination.

Müller and Lindenberger (2011), in coupling respiration among participants, likewise registered synchronization in heart rate variability. Relative phase synchronization in heart beats is exhibited between co-sleepers in a bi-directional fashion (Yoon et al., 2019). The inclusion of cognitive measures in such paradigms produces an intra- and inter-individual relationship between physiological variables and their associated cognitive activity. Murata found individuals’ subjective excitement in a cooperative game increased not only with their own heart rate, but also with their partner’s (Murata et al., 2021). Observing bystanders’ reports of perceived excitement increased with players’ heart rate synchrony. Kang and Wheatley’s group pupillometry demonstrated a synchronization of pupil dilation in expressive speakers and empathetic listeners (Kang and Wheatley, 2017). Pupillary synchrony was strongest at emotional peaks and less engaging moments observed a diminished coupling.

An entire class of experiments can be designed for the extended study of resonant combination through the shared resonance of non-neural endogenous rhythms concomitant with measures of cognitive activity. An alternative path can be the addition of hyperscanning physiological measures to pre-existing paradigms starting with the previous two studies proposed. It may be hypothesized that, in line with Murata and Kang’s separate findings, synchrony in the physiological markers, similar to INS, will be associated with some manner of merging experience. In time, a comprehensive framework encompassing neural, behavioral, physiological, and other shared resonances can be developed detailing the multimodal process of resonant combination.

## Conclusion

The present paper offers a reanalysis of the literature and proposes, through our model of interpersonal resonant combination, an explanation of INS-driven micro-to-macro consciousness merging toward a new and higher-level phenomenal consciousness. Entraining behavioral rhythms, selected for by mutual attention, appear to underlie the coupling of rhythmic neural oscillations between discrete brains. Syllabic rate of speech, respiratory cycles, interpersonal sexual activity, and behavioral cue exchange have been explored as possible common denominator rhythms that demarcate the boundaries of the coupled, cognitive system. Phase transitions, the critical point marking combination, are hypothesized to be represented in increased cooperative ability and altered states of consciousness that are associated with INS establishment. It is our hypothesis that the current literature supports a capacity for interpersonal resonant combination.

Empathetically related groups engaged in joint action provide the greatest potential for such a phenomenon. However, autism spectrum disorder (ASD), characterized by atypical social interaction and communication, has been linked to a decreased capacity to synchronize neural activity with communicative partners (Quiñones-Camacho et al., 2021). The coupling deficit is extended to include interpersonal motor, conversational, and physiological synchrony (McNaughton and Redcay, 2020). These recent findings yield a new dimension to the resonant combination model. Although it can be concluded with ease that empathetically related groups sharing attention possess the greatest potential for resonant combination, the conclusion may require a future addendum to further state: “empathetically related groups *of neurotypical individuals* engaged in joint action possess the greatest potential for resonant combination.” Subsequent research will decide the matter of its inclusion.

ASC mapping of individuals pre-, peri-, and post-combination provides an approach appropriate for the current state of cognitive neuroscience. To this end, we suggest three avenues of empirical exploration for exploring combined consciousness. Results are hypothesized to represent a convergence of experience between synchronized individuals.

In sum, our proposed model constitutes the first step in a million-mile journey toward generating a concrete account of micro-to-macro combination beyond discrete brains. There is a general shift, beginning with the oscillatory correlates approach (Young, 2022, in press), toward a greater consideration of supervening cognitive systems and our capability for interpersonal merging. Containing two decades of INS literature we have described what is known, identified what is missing, and speculated on what is yet to be discovered. It is with great confidence and dense empirical evidence that we theorize the innate potential for a consciousness transcending the complex subjective experience we now enjoy.

## Data Availability Statement

The original contributions presented in the study are included in the article/supplementary material, further inquiries can be directed to the corresponding author.

## Author Contributions

AY designed the model. AY, IR, and SS conducted the literature reviews and wrote the paper. All authors contributed to the article and approved the submitted version.

## Conflict of Interest

The authors declare that the research was conducted in the absence of any commercial or financial relationships that could be construed as a potential conflict of interest.

## Publisher’s Note

All claims expressed in this article are solely those of the authors and do not necessarily represent those of their affiliated organizations, or those of the publisher, the editors and the reviewers. Any product that may be evaluated in this article, or claim that may be made by its manufacturer, is not guaranteed or endorsed by the publisher.
